# Agreement Between Swept-source Optical Coherence Tomography and Rotating Scheimpflug Camera in Measurement of Corneal Parameters in Normal and Keratoconic Eyes

**DOI:** 10.18502/jovr.v18i4.14547

**Published:** 2023-11-30

**Authors:** Hamed Ghassemi, Mehran Zarei-Ghanavati, Mina Khastavan, Mehrnaz Atighehchian, Abbas Azimi Khorasani, Golshan Latifi

**Affiliations:** ^1^Farabi Eye Hospital, Tehran University of Medical Sciences, Tehran, Iran; ^2^Refractive Error Research Center, Department of Optometry, Mashhad University of Medical Sciences, Mashhad, Iran; ^4^Hamed Ghassemi: https://orcid.org/0000-0001-9048-6726; ^5^Mehrnaz Atighehchian: http://orcid.org/0000-0002-6357-1033C

**Keywords:** Optical Coherence Tomography, Keratoconus, Pentacam, CASIA2

## Abstract

**Purpose:**

This study aimed to assess the agreement between topographic indices of healthy subjects and keratoconus (KCN) patients using a swept-source optical coherence tomography (SS-OCT CASIA2) versus a Scheimpflug camera (Pentacam).

**Methods:**

40 eyes of 23 patients with KCN and 40 eyes of 20 healthy subjects were included and evaluated with the CASIA2, followed by the Pentacam. Two consecutive modalities were obtained for one eye of each patient. Corneal parameters, including anterior keratometry at steep (Ks) and flat meridians (Kf), anterior astigmatism, anterior and posterior corneal elevation values, thinnest corneal thickness, and apex corneal thickness, were evaluated.

**Results:**

CASIA2 and Pentacam showed perfect agreement (95% limits of agreement (LoA): -0.22 to 0.68, 95% LoA: -1.5 to 1.44 D) and good correlation (Intraclass correlation (ICC):0.986, ICC:0.987; P< 0.01) for anterior (Ks) in normal and ectatic corneas, respectively. The cylinder amount had moderate agreement and correlation (95% LoA: -0.55 to 0.47D, ICC: 0.797, P< 0.01) in normal, and moderate to strong agreement and correlation (95% LoA: -1.57 to 0.87D, ICC=0.911, P< 0.01) in Keratoconic eyes. There was a fair agreement for anterior and posterior corneal elevation values in normal subjects (95% LoA: -3.09 to 4.59, 95% LoA: -6.91 to 7.31D). The thinnest corneal thickness amount had an excellent agreement in normal and KCN patients (ICC: 0.983, 0.953; respectively).

**Conclusion:**

Although the devices had different mean indices values, they had a good agreement based on the Bland–Altman plots. Since Pentacam is accepted as the standard tool for diagnosing ectatic cornea, pentacam CASIA2 is also helpful for early diagnosis of KCN.

##  Introduction

Topographical evaluation of the corneal configuration is necessary for detecting corneal abnormality before refractive and cataract surgery. Specific geometric parameters, including asphericity, corneal curvature, and thickness values, provide different analyses for detecting early corneal abnormalities^[1,2]^. To prevent refractive surgery complications, precise preoperative evaluation is necessary to diagnose subclinical keratoconus (KCN) as the main cause of ectasia^[2,3]^. In addition, indications for cornea cross-linking in advanced KCN depend on the accurate measurement of corneal topography variation^[4]^. Several sophisticated devices have been employed, such as scanning-slit topography, Scheimpflug imaging, and optical coherence tomography (OCT)^[5,6,7]^.

Scheimpflug imaging, like the Pentacam, provides numerous images to evaluate the anterior and posterior corneal surfaces as well as to remodel the overall corneal profile ^[7]^. Based on the Scheimpflug concept, the maximum depth of focus with the least image distortion^[8]^ is documented. Also, three-dimensional anterior segment OCT(AS-OCT), like CASIA, is based on swept-source Fourier-domain mechanics and is one of the newest methods to diagnose KCN eyes in the early stage^[9]^. The light source of CASIA has a central wavelength of 1.3 mm and a speed of 30,000 A-lines/seconds. This device can analyze anterior and posterior cornea curvature, topography and pachymetry mapping, and regular and irregular corneal astigmatism, with faster parameters acquisition and greater repeatability and reproducibility than Scheimpflug imaging^[7,8,9,10,11]^.

Some studies have attempted to compare the precision of three-dimensional AS-OCT CASIA (SS-1000) with Scheimpflug imaging in KCN eyes. However, there is no report of a correction formula to convert CASIA2 parameters to Pentacam to modify the parameter differences. This study compared the calculated topographic parameters agreement between CASIA2 and Pentacam in KCN patients and normal subjects.

##  Methods

This study was conducted at the Refractive Surgery Clinic of Farabi Eye Hospital, Tehran University of Medical Sciences. This study adhered to the tenets of the Declaration of Helsinki, and the protocol of the study was approved by the Ethics Committee of Mashhad University of Medical Sciences, Mashhad, Iran, and the approval number was Ir.mums.rec.1398.241. Informed consent was obtained from all participants.

The study included 40 normal and 40 KCN participants between the ages of 21 and 38. According to topographic parameters and clinical slit-lamp biomicroscopy, keratoconus eyes were classified into mild, moderate, and severe.

The sample size was chosen according to this statistical formula: 


$$n=\frac{{\left(Z_{1-\alpha /2}+Z_{1-\beta }\right)}^{2}\times \left(S_{1}^{2}+S_{2}^{2}\right)}{d^{2}}$$

α =type1 errors50%

β =power test80%

S1 = pachymetry thinnest point standard deviation in normal cases

S2 = pachymetry thinnest point standard deviation in KCN cases

d =difference

n =sample size

The inclusion criteria for healthy participants were normal topography, slit-lamp biomicroscopy and the best spectacle-corrected visual acuity (BCVA) of 20/20 or better. The inclusion criteria for KCN patients were at least one clinical keratoconus sign in slit-lamp bio-microscopy including Vogt's striae، Fleischer's ring، thinning at apex, Rizutti's sign, and topography index (skewed, asymmetric bow-tie, inferior steepening), corneal curvature of more than 47.2 D, inferior-superior (I-S) value of more than 1.4 D, nipple, oval, or globus cone appearance in the Pentacam.

Exclusion criteria were advanced KCN, contact lens wearers (rigid contact lens and soft contact lens of more than 4 to 2 weeks, respectively), previous corneal cross-linking, corneal ring implantation, previous keratoplasty, and refractive surgery.

All patients underwent visual acuity measurement and complete ophthalmic examination with slit-lamp bio-microscopy. Topographic evaluations were performed with the SS-OCT (CASIA2, Tomey Corp, Japan) and the Scheimpflug camera (Pentacam HR, Oculus, Wetzlar, Germany) imaging. The keratoconus was graded based on the previous grading to mild (mean K < 47.0 D) and moderate (mean K 47.0 to 52.0 D). We selected some parameters related to the anterior surface to assess the agreement between CASIA2 and Pentacam, including anterior keratometry at steep (Ks)and flat meridians (Kf), anterior astigmatism values, thinnest corneal thickness (TCT) and corneal thickness at the apex(ACT) and anterior and posterior highest points (AHP, PHP) values.

**Figure 1:**
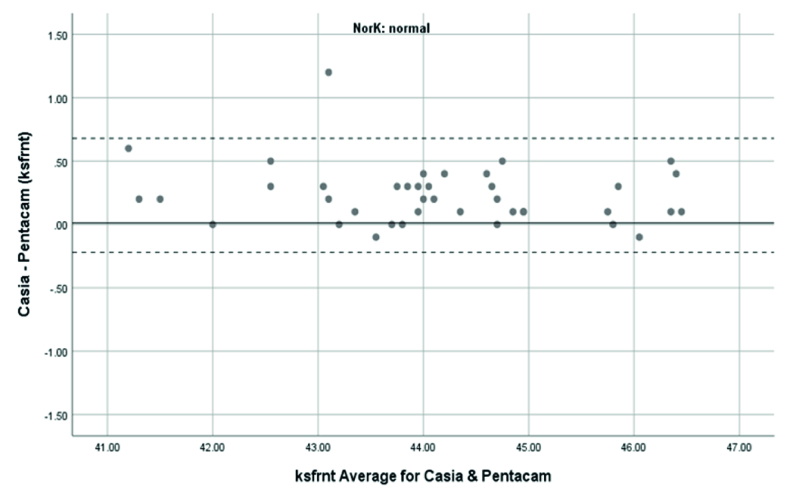
Bland-Altman plot shows the agreement anterior Ks measurements between SS-OCT(CASIA2) and Scheimpflug camera(Pentacam) in normal subjects. K, keratometry; Ksfrnt, anterior keratometry at seep meridian.

**Figure 2:**
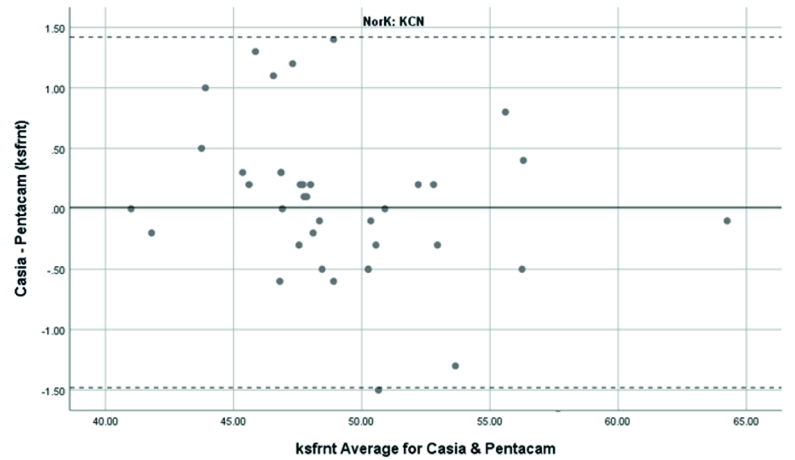
Bland-Altman plot shows the agreement in anterior Ks measurements between SS-OCT(CASIA2) and Scheimpflug camera(Pentacam) in keratoconus cases. KCN, Keratoconus; Ksfrnt, anterior keratometry at seep meridian.

**Figure 3:**
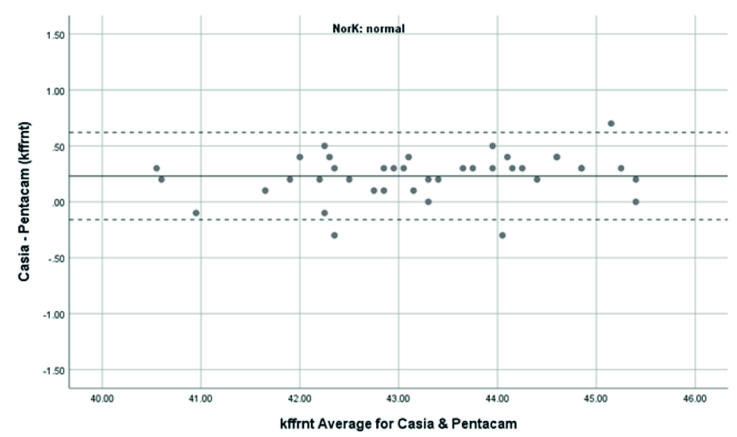
Bland-Altman plot shows the agreement in anterior Kf measurements between SS-OCT(CASIA2) and Scheimpflug camera(Pentacam) in normal cases. kffrnt, anterior keratometry at flat meridian.

**Figure 4:**
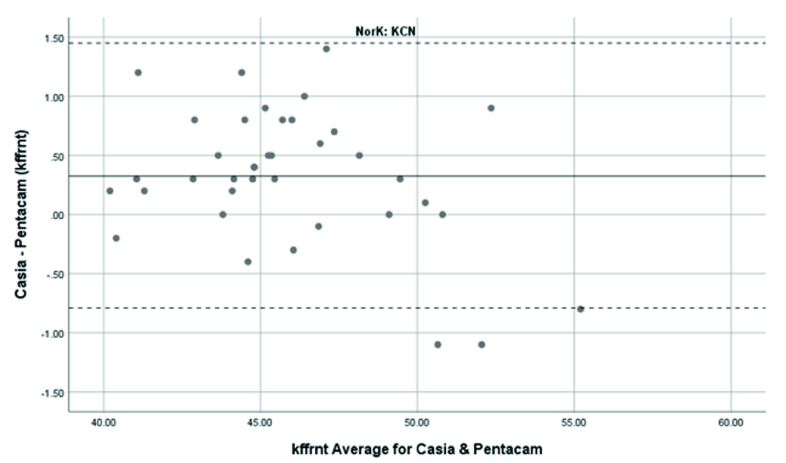
Bland-Altman plot shows the agreement in anterior Kf measurements between SS-OCT(CASIA2) and Scheimpflug camera(Pentacam) in keratoconus cases. KCN, Keratoconus; Kffrnt, anterior keratometry at flat meridian.

**Figure 5:**
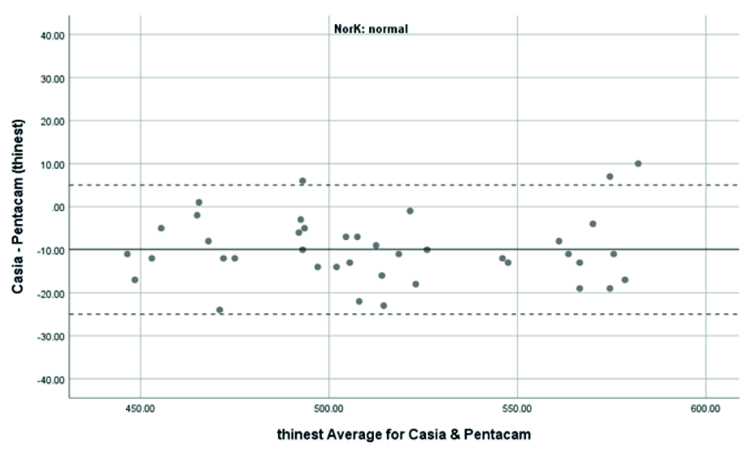
Bland-Altman plot shows the agreement in TCT measurements between SS-OCT(CASIA2) and Scheimpflug camera(Pentacam) in the normal case.

**Figure 6:**
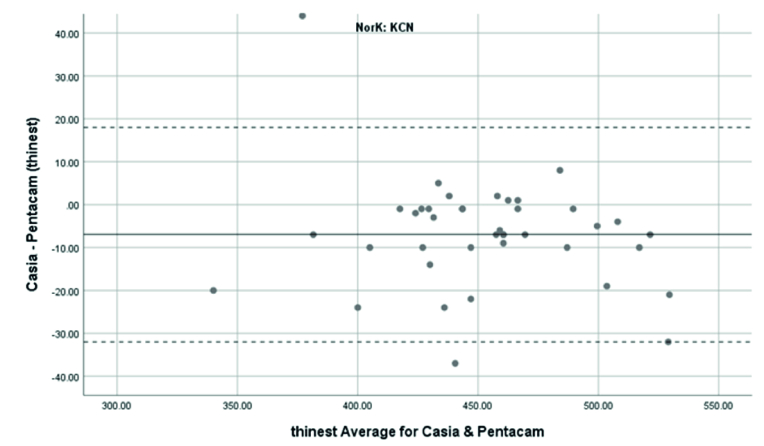
Bland-Altman plot shows the agreement in TCT measurements between SS-OCT(CASIA2) and Scheimpflug camera(Pentacam) in keratoconus cases. KCN, Keratoconus.

**Figure 7:**
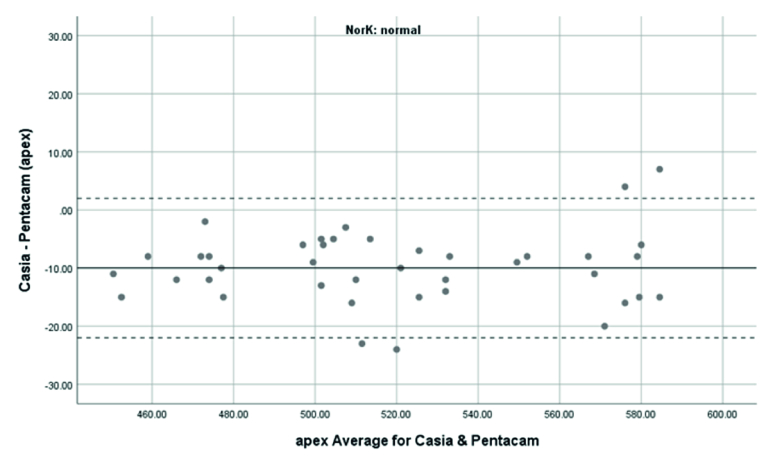
Bland-Altman plot showing the agreement in ACT measurements between SS-OCT and Scheimpflug camera in normal cases.

**Figure 8:**
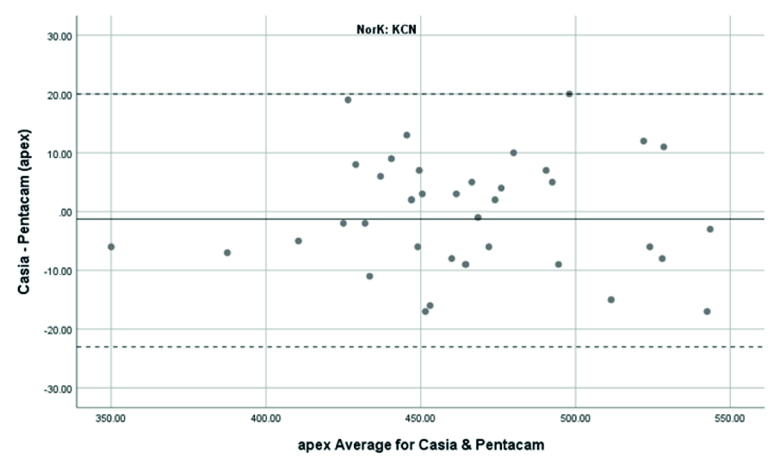
Bland-Altman plot shows the agreement in ACT between SS-OCT(CASIA2) and Scheimpflug camera(Pentacam) in keratoconus cases. KCN, Keratoconus.

**Table 1:** sex and age distribution.


	orangeSex/male	orangeMean age/year
Normal	10(25%)	30.75 ± 3.6
Mild KCN	4(25%)	29.31 ± 4.7
Severe KCN	7(31.8%)	27.18 ± 5.0
	
	

**Table 2:** Parameters in normal patients.


orangeParameter	orangeCASIA2 Mean ± SD	orangePentacam Mean ± SD	orangeDifference 95%lower/upper	orangeICC 95%lower/upper	orange95%LOA Lower/upper	oranger	orangeBeta0	orangeBeta1
Anterior Ks	44.25 ± 1.37	44.02 ± 1.41	0.23 ± 0.23 (0.16to0.3)	0.986(0.973 to 0.992)	-0.22to 0.68	0.986*	-0.97	1.02
Anterior Kf	43.39 ± 1.31	43.16 ± 1.26	0.23 ± 0.29 (0.17to0.29)	0.988(0.977 to 0.994)	-0.16to 0.62	0.989*	1.81	0.95
Anterior corneal cylinder	0.84 ± 0.35	0.87 ± 0.44	-0.04 ± 0.26 (-0.12to 0.05)	0.797(0.648 to 0.887)	-0.55 to 0.47	0.817*	0.01	1.03
TCT	509 ± 42	519 ± 41	-9.88 ± 7.74 (-12.35to -7.4)	0.983(0.967 to 0.991)	-25.05 to 5.25	0.983*	22.37	0.98
ACT	515 ± 41	525 ± 41	-9.98 ± 6.13 (-11.94 to -8.01)	0.989(0.979to0.994)	-21.99 to 2.03	0.989*	21.48	0.98
AHP	5.0 ± 2	4.0 ± 2	0.75 ± 1.96 (0.12to 1.38)	0.302(-0.006 to 0.558)	-3.09 to 4.59	0.303	2.82	0.28
PHP	11 ± 5	11 ± 5	0.2 ± 3.63 (-0.96to1.36)	0.692(0.488 to 0.824)	-6.91 to 7.31	0.693*	2.76	0.73
	
	
white<bcol>9</ecol>Ks, keratometry at seep meridian; Kf, keratometry at flat meridian; SD,standard deviation; LoA, limit of agreement based on linear mixed model analysis; ICC, Intraclass correlation; TCT, thinnest corneal thickness; ACT, apex corneal thickness; AHP, Anterior highest point (anterior surface elevation); PHP, Posterior highest point (posterior surface elevation); Beta0, Intercept of correction formula based on linear regression; Beta1, The correction coefficient based on linear regression; r, Pearson Correlation Coefficient relation. * P< 0.01

**Table 3:** Parameters in KCN patients.


orangeParameter	orangeCASIA2 Mean ± SD	orangePentacam Mean ± SD	orangeDifference 95%lower/upper	orangeICC 95%lower/upper	orange95%LOA Lower/upper	oranger	orangeBeta0	orangeBeta1
Anterior Ks	49.03 ± 3.74	49.06 ± 4.06	-0.03 ± 0.75 (-0.27 to 0.21)	0.987(0.975 to 0.993)	-1.5to1.44	.989**	-2.47	1.05
Anterior Kf	46.05 ± 3.32	45.73 ± 3.52	0.33 ± 0.57 (0.14 to 0.51)	0.986(0.974 to 0.997)	-0.79to1.45	.865**	-13.08	1.29
Anterior corneal cylinder	2.95 ± 1.4	3.31 ± 1.52	-0.35 ± 0.62 (-0.55 to -0.15)	0.911(0.835 to0.952)	-1.57to0.87	.889*	-2.46	2.36
TCT	449 ± 41	456 ± 43	-6.95 ± 12.91 ( -11.08 to -2.82)	0.953(0.913 to 0.975)	-32.25to18.35	.955**	0.36	1.01
ACT	464 ± 41	465 ± 41	-1.28 ± 10.96 (-4.78 to 2.23)	0.965( 0.934 to0.981)	-22.76to20.2	.965**	19.23	0.96
AHP	19 ± 12	22 ± 12	-2.18 ± 4.7 (-3.73 to -0.64)	0.913(0.840 to 0.953)	-11.39to7.03	.915**	2.22	0.98
PHP	41 ± 22	48 ± 24	-6.38 ± 10.25 (-9.71 to -3.06)	0.903(0.822 to 0.948)	-26.47to13.71	.905**	7.49	0.97
	
	
white<bcol>9</ecol>Ks, keratometry at seep meridian; Kf, keratometry at flat meridian; SD, standard deviation; LoA, limit of agreement based on linear mixed model analysis; ICC, Intraclass correlation; TCT, thinnest corneal thickness; ACT, apex corneal thickness; AHP, Anterior highest point (anterior surface elevation); PHP, Posterior highest point (posterior surface elevation); Beta0, Intercept of correction formula based on linear regression; Beta1, The correction coefficient based on linear regression; r, Pearson Correlation Coefficient relation * P< 0.05; ** P< 0.01

### Swept-source OCT

CASIA2 is a swept-source AS-OCT that captures three-dimensional images with a wavelength of 1310 nm and measures with a very fast scanning speed (50.000 A-scan/sec). The scan time is 0.3 seconds to estimate corneal topography and thickness value with 11 mm scan depth. This device can evaluate anterior and posterior corneal surfaces to assess corneal power, anterior and posterior elevation values, and corneal thickness^[6,7]^.

### Scheimpflug imaging

Pentacam Scheimpflug Imaging (Pentacam HR, Oculus, Germany) has a rotating Scheimpflug camera to obtain 50 slit images with a slit depth of 14.0 mm in less than two seconds. Each image has 500 true elevation points for a total of 25,000 true points^[1,7]^.

### Statistical analysis 

To present statistics, we used mean and standard deviation ranges. To compare the two sets, we used a linear mixed model. In the construction of the limit of agreement (LoA), we also utilized the linear mixed-effects model analysis in the mentioned evaluations (Parker RA, Scott C, Inácio V, Stevens NT. Using multiple agreement methods for continuous repeated measures data: a tutorial for practitioners. BMC Med Res Methodol. 2020; 20:154). We used ICC (intraclass correlation) and r (Pearson Correlation Coefficient relation) to present the similarity of the findings. To present the correction formula to transform values from CASIA2 to Pentacam, we used linear regression analysis and the following formula: Pentacam _value = Beta0 + Beta1 * CASIA2_value. The correction ability of these formulae is presented by Pearson correlation coefficients.

##  Results

40 eyes of 23 keratoconus patients and 40 eyes of 20 healthy participants were included in this study. KCN was mild in 16 eyes and was moderate in 22 eyes. Two eyes with severe KCN were excluded. All patients aged between 21 and 38 years. Mean ages in normal subjects and mild and moderate KCN patients were 30.75 ± 3.6, 29.31 ± 4.7, and 27.18 ± 5.0 years, respectively. In the normal group, ten eyes (25%); in the mild KCN group, four eyes (25%); and in the moderate group, seven eyes (31.8%) were male (Table 1). There was no significant statistical discrepancy between sex and age in the normal participants and KCN patients.

### Agreement of anterior keratometry measurements

Anterior Ks in the normal subjects were measured using the Pentacam and CASIA2 with a resultant mean of 44.02 ± 1.41 and 44.25 ± 1.37 D, respectively. The range of 95% LoA was -0.22 to 0.68D. Therefore, it showed an excellent agreement and correlation between the Pentacam and CASIA2 (ICC: 0.986 with 95% CI: 0.973 to 0.992D, r =0.986, P< 0.01).

Anterior Ks in KCN patients were measured using the Pentacam and CASIA2 with a mean of 49.06 ± 4.06 and 49.03 ± 3.74 D, respectively. The 95% LoA between the Pentacam and CASIA2 was -1.5 to 1.44D. So, it showed an excellent agreement and correlation between the Pentacam and CASIA2 (ICC: 0.987 with 95% CI: 0.975 to 0.993D, r =0.989 P< 0.01).

### Agreement of anterior corneal cylinder

In the normal group, the range of 95% LoA was -0.55 to 0.47D. Hence, there was moderate agreement and correlation between the Pentacam and CASIA2(ICC: 0.797 with 95%CI 0.648 to 0.887D, r=0.817, P< 0.01). However, in the KCN group, this index reflected good agreement and correlation (ICC: 0.911 with 95% CI:0.835 to 0.952, r=0.889) between the Pentacam and the CASIA2.

### Agreement of anterior highest point (anterior surface elevation)

In the normal group, the range of 95% LoA was -3.09 to 4.59 mm. So, there was fair agreement and correlation between the Pentacam and CASIA2 (ICC: 0.302 with 95%l:0.006 to 0.558, r =0.303, P< 0.01). However, in the KCN group, this index demonstrated excellent agreement and correlation (ICC:0.913 with 95% CI: 0.840 to 0.953, r=0.915).

### Agreement of TCT Measurements

TCT in normal subjects was measured using the Pentacam and CASIA2 with a mean ± SD of 519 ± 41 μm, 509 ± 42 μm. In the KCN patients, it was 456 ± 43 μm and 449 ± 41 μm, respectively. TCT measurement with the Pentacam was thicker than those analyzed with the CASIA2. The difference was 9.88 ± 7.74 μm (95% CI: 12.35 to 7.4) in normal subjects and was 6.95 ± 12.91 μm (95%CI: -11.08 to -2.82) in the KCN group, but there was no significant difference between the two devices ( P> 0.05). The correction formula to transform values from the CASIA2 to the Pentacam was Beta0=22.37 and Beta1=0.98 in the normal group and Beta0=-2.47 and Beta1=1.05 in the KCN group.

The 95% LoA between the Pentacam and CASIA2 in the measurement of the TCT was -25.05 to 5.29 μm in the normal group and -32.25 to 18.35 μm in the KCN group. These results showed significant correlations between the Pentacam and the CASIA2 in the normal (r =0.983 ICC: 0.983) and KCN groups (r =0.955, ICC: 0.953).

### Agreement of ACT Measurements

The mean ACT measurement in normal subjects was 525 ± 41 μm with the Pentacam and 515 ± 41 μm with the CASIA2. The corresponding figures were 456 ± 43 μm and 449 ± 41 μm in the KCN group, respectively. ACT measurement with the Pentacam was thicker than those evaluated with the CASIA2. The difference was 9.98 ± 6.13 μm in the normal and 1.28 ± 10.96 μm in the KCN groups, but there was no significant difference between the two devices( P> 0.05). The correction formula to transform values from the CASIA2 to the Pentacam was Beta0= 21.48 and Beta1=0.98 in the normal and Beta0= 19.23 and Beta1=0.96 in the KCN groups. The 95% LoA of the Pentacam with CASIA2 in the measurement of the ACT was -21.99 to 2.03 μm in the normal and -22.76 to 20.02 μm in the KCN groups. These results showed significant correlations between the Pentacam and CASIA2 in the normal subjects (r =0.989; ICC: 0.989) and the KCN patients (r =0.965; ICC: 0.965).

Tables 2 and 3 summarize all data measurements and limits of agreement between the Pentacam and CASIA2.

Overall, anterior steep and flat cornea keratometry showed perfect and strong agreement in the results for normal and ectatic corneas; Bland–Altman plots of agreement in corneal indices between the CASIA2 and Pentacam are demonstrated in Figures 1 to 8.

##  Discussion

It is essential to compare new techniques for evaluating corneal topography to determine the agreement between several instruments^[7,12]^. Dissimilar results of the same corneal parameters from multiple devices can affect the diagnosis a corneal disease like keratoconus^[13]^. In this cross-sectional study, we analyzed corneal parameters using Scheimpflug–Placido (Pentacam) versus swept-source OCT (CASIA2) to compare the keratoconus patients and normal subjects to determine the agreement of the most important and diagnostic indices.

OCT works based on measuring delay in light back-scattered from various surface depths. Swept Source Fourier-domain OCT is a recent method combining light beam reflections and reference arms. This technology is much faster than conventional OCT with a higher axial and lateral resolution, so it can decrease motion artifacts for scanning larger depths of focus^[7,14]^. Moreover, the Fourier-domain OCT system uses a corneal pachymetry map to acquire corneal thickness measurements and provides high accuracy in keratoconus detection^[15,16,17,18]^.

The Scheimpflug principle explains optical properties when the three main planes of film, lens and focus are not aligned with each other^[7]^. The Pentacam-Scheimpflug is a non-contact instrument with a rotating Scheimpflug camera that takes up to 50 slit images in less than two seconds^[12]^.

In the normal and KCN groups, we detected perfect agreement between the Pentacam and CASIA2 in calculating anterior Ks and Kf indices, similar to previous reports^[7,9,11]^. A previous study^[11]^ compared SS-OCT with dual rotating Scheimpflug and Placido–scanning–slit systems in normal and post-refractive patients. In their result, the anterior keratometry calculation showed a perfect agreement among the three systems. The calculation of posterior keratometry showed an excellent agreement between the dual rotating Scheimpflug–Placido system and SS-OCT, but this correlation was not detected in the Placido–scanning-slit system. Eszter et al.^[10]^ found significant differences in keratometry measurements between the CASIA SS-1000 and Pentacam. However, the repeatability of their measurements was near and comparable.

In evaluating corneal thickness, we found a perfect agreement between the two devices in both normal subjects and KCN patients. We compared quantitative agreement by calculating the 95% LoA and correlation by ICC. The 95% LoA ranged in normal subjects, from -25.05 to 5.25 and -21.99 to 2.03 μm in the TCT and ACT calculations, respectively. The observed 95% LoA ranged from -32.25 to 18.35 for TCT and -22.76 to 20.2 μm for ACT in keratoconic eyes. This finding resembles what previous studies found regarding the average thickness measurements^7,1516^. Ghoreishi et al.^[7]^ investigated the thickness parameters (central, thinnest, and apex thicknesses) and the correlation between the CASIA SS-100 and Pentacam in normal and keratoconus patients. Their study showed good agreement.

Akihiro et al.^[15]^ found a high correlation between FD-OCT (RTVue-100), ultrasonic pachymetry, and Pentacam in central corneal thickness (CCT) measurements. The OCT underestimated the CCT thickness as compared with the Pentacam. We found slightly thinner means of TCT, ACT in normal (9.88, 9.97 μm) and KCN (6.95, 1.28 μm) cases in CASIA2 as compared to the Pentacam, that were not statistically significant ( P> 0.05). There were similar calculations of TCT and ACT in the normal (ICC: 0.983, r=0.983 and 0.989, r=0.989) and in the KCN groups (ICC: 0.953, r=0.955 and 0.965 r=0.965) suggesting a good correlation between CASIA2 and Pentacam for TCT and ACT.

We could not find any report that presented a correction formula. Our study analyzed the differences between all measurements and suggested a correction formula to convert the CASIA2 parameters to the Pentacam parameters to modify the parameter differences.

Ghoreishi et al.^[7]^ also found agreement in all means of the highest or lowest data points except in the posterior lowest point in KCN patients. In our study, in the KCN group, anterior and posterior surface elevation values had an excellent agreement with a 95% LoA -11.39 to 7.03 and -26.47 to 13.71, respectively. In contrast, in the normal group, these indices had a fair agreement with a narrow range of LoA.

Some limitations must be acknowledged. The major limitation of this study was the small sample size, limited evaluated parameters, and a lack of a repeatability analysis. Therefore, it is necessary to introduce complete data to support our conclusions in further studies. Our research supports the results of previous studies on two systems of Scheimpflug and SS-OCT regarding the variables of anterior and posterior keratometry indices, ACT and TCT. On the other hand, our study suggested applying a new correction formula to convert CASIA2 parameters to Pentacam for correcting parameter differences and using the parameters interchangeably and as an alternative.

In summary, our research supports the results of previous studies and shows that the CASIA2 is a reliable device for corneal and anterior segment biometry in the normal and ectatic cornea. Therefore, it can be used interchangeably with the Pentacam. It may be a useful alternative for measuring steep and flat anterior keratometry, TCT, and ACT. However, in normal subjects, fair agreement in anterior and posterior corneal curvature values and moderate agreement in the anterior corneal cylinder were detected. So, it requires more supplementary assessments. This study also presents a new formula to change findings from the CASIA2 to Pentacam for correcting the indices difference. Since the Pentacam is accepted as the standard tool for diagnosing ectatic cornea, compared to the Pentacam, CASIA2 is similar to a useful diagnostic tool for the early diagnosis of KCN.

##  Financial Support and Sponsorship

None.

##  Conflicts of Interest

None.
